# Antioxidant and Antifibrotic Effect of a Herbal Formulation *In Vitro* and in the Experimental Andropause via Nrf2/HO-1 Signaling Pathway

**DOI:** 10.1155/2017/6024839

**Published:** 2017-09-17

**Authors:** Woong Jin Bae, Guan Qun Zhu, Sae Woong Choi, Hyun Cheol Jeong, Fahad Bashraheel, Kang Sup Kim, Su Jin Kim, Hyuk Jin Cho, U Syn Ha, Sung Hoo Hong, Ji Youl Lee, Hyun-A Oh, Hye Cheong Koo, Do Ram Kim, Sung Yeoun Hwang, Sae Woong Kim

**Affiliations:** ^1^Catholic Integrative Medicine Research Institute, College of Medicine, The Catholic University of Korea, Seoul, Republic of Korea; ^2^Department of Urology, College of Medicine, The Catholic University of Korea, Seoul, Republic of Korea; ^3^KEMIMEDI, Seoul, Republic of Korea

## Abstract

The Korean herbal formulation Ojayeonjonghwan is used for improving late-onset hypogonadism (LOH) symptoms such as erectile dysfunction (ED). A previous research suggested that a modified Ojayeonjonghwan (KH-204) could be used as an alternative to the treatment for ED. The pharmacological effects were examined in different conditions, including in vitro and in vivo. We measured the survival rate of TM3 Leydig cells under the oxidative stress condition. The s.c. injection of leuprorelin was used to induce androgen deprivation. We measured serum testosterone levels, oxidative stress, and apoptosis. The results of the treatment by KH-204 (1) preserved TM3 cells from oxidative stress by improving the expression of nuclear factor erythroid 2-related factor 2 (Nrf2)/heme oxygenase-1 (HO-1); (2) lowered the expression of transforming growth factor-beta (TGF-*β*) 1/SMAD; (3) increased the average of serum testosterone in androgen-deprived male rats; (4) kept the activation of spermatogenesis; (5) upgraded the contents of 8-hydroxy-20-deoxyguanosine (8-OHdG) and degraded the contents of superoxide dismutase (SOD); and (6) reduced apoptosis. We studied that KH-204 improved testicular dysfunction in LOH. It is likely, at least in part, to degrade oxidative stress through the Nrf2/HO-1 pathway. These findings may offer credible evidences for the use of new alternative therapies to treat LOH.

## 1. Introduction

Late-onset hypogonadism (LOH) includes symptoms such as depression, fatigue, low muscle mass, and erectile dysfunction (ED) and occurs in middle-aged males. Many studies have demonstrated that LOH is closely linked to a deficiency in serum testosterone levels [1–3] because the total number of Leydig cells, which are the main source of testosterone in middle-aged men, decreases to about a half of that seen in young men [4, 5]. LOH symptoms are associated with testosterone deficiency and, therefore, can be improved by androgen replacement therapy (ART). ART can be used by men with LOH unless there are contraindications such as unstable cardiovascular disease, prostate cancer, or polycythemia; however, the long-term effects of ART are uncertain. In particular, external testosterone supplementation can contemporaneously increase the incidence of side effects including prostate cancer, benign prostatic hyperplasia (BPH), and cardiovascular events [5, 6]. ART can impair fertility, so men who would like to start a family in the short run should make sure that they should not undergo ART [7].

Traditional herbal medicine is recognized as an alternative treatment of LOH that avoids the adverse effects of ART and, in some cases, can be of benefit to libido [8, 9]. Now, male infertility and LOH symptoms including ED are usually treated by the herbal formulation Ojayeonjonghwan [10, 11]. Park et al. [10] illuminated that improved Ojayeonjonghwan, known as KH-204, ameliorated ED caused by peripheral neuropathy in aged and diabetic rats. Recently, we also found that KH-204 protected against oxidative stress in rat testis [12]. A major characteristic of aging is the increased oxidative stress. It has been involved in various age-related pathologies [13]. Regarding interacting proteins and regulatory molecules such as heme oxygenase-1 (HO-1), the nuclear factor erythroid 2-related factor 2 (Nrf2) signaling system has become the most significant cellular defense mechanism against oxidative stress, and many reports suggest that the reduction in the adaptive response of the Nrf2 signaling system has had a great influence in the cumulation of oxidative damage in aging [14–16].

Some previous studies demonstrated that progressive fibrosis was recognized as a characteristic in various organs, playing an important role during aging [17, 18]. Transforming growth factor-beta (TGF-*β*) is considered to have a significant effect in the pathogenic mechanism of diabetes-induced ED. Especially, TGF-*β*1 has been regarded as one of the most relevant fibrogenic cytokines, which is also high expression in the corpus cavernosum of diabetic rats [19]. Zhang et al. [20] also considered that upregulation of the TGF-*β*1/SMAD signaling pathway led to structural changes and decline of erectile function in ED. However, the impact of TGF-*β*1/SMAD signaling pathway on Leydig cells including testicular function is still unidentified.

The present study was intended to assess whether KH-204 could protect TM3 Leydig cells through antioxidant activity in vitro and restore testosterone production in an androgen-deprived animal model. In addition, we attempted to identify a potential mechanism of the protective effect.

## 2. Materials and Methods

### 2.1. Preparation of the Herbal Formula (KH-204)

The major ingredients of KH-204 include five plants, as previously described: *Cornus officinalis* (32%), *Lycium chinense* (32%), *Rubus coreanus* (16%), *Cuscuta chinensis* (16%), and *Schisandra chinensis* (4%). It was manufactured by a company that makes oriental herbal medicines, KEMIMEDI Co. Ltd. (Seoul, Republic of Korea). The quality of KH-204 was confirmed by its marker compound. In addition, the quality of each extract were identified by using high-performance liquid chromatography (HPLC) as previously described [21]. The marker compounds of *Cornus officinalis*, *Lycium chinense*, *Rubus coreanus*, *Cuscuta chinensi*, and *Schisandra chinensis* are loganin, betain, ellagic acid, hyperoside, and Schizandrin in HPLC chromatogram, respectively. The manufacturing method and the toxicity data of KH-204 were described in a previous report [22].

### 2.2. *In Vitro* Cell Viability and Western Blot Testing Using TM3 Mouse Leydig Cells

TM3 mouse Leydig cells (Korean Cell Line Bank, Seoul, Republic of Korea) were cultured in Dulbecco's modified eagle's medium (DMEM)/F-12 medium (GIBCO®, Life Technologies Co., USA) supplemented with 10% heat-inactivated fetal bovine serum (FBS; GIBCO) at 37°C. Cells were plated on 96-well plates (Corning) in 10% FBS/DMEM/F-12 and incubated for twenty-four hours. They were pretreated with 50 *μ*g/ml of KH-204 for two hours before treating with hydrogen peroxide (40 *μ*M H_2_O_2_) for two hours to create oxidative cellular stress. Afterwards, Alamar Blue (Invitrogen, USA) was aseptically added to the cells. The cells were incubated for three hours, and the absorbance of the cells was measured at a wavelength of 570 nm using an enzyme-linked immunosorbent assay (ELISA) reader (Molecular Devices, USA). The background absorbance was measured at 600 nm and was then subtracted. Cell viability treated by ERK inhibitor PD98059 or Akt inhibitor LY294002 was also assessed to identify the activation of ERK and Akt.

After processing, we gathered all cellular proteins, by placing cells in a lysis buffer consisting of 0.1% sodium dodecyl sulfate in phosphate-buffered saline, followed by brief sonication. Protein concentration was identified by a bicinchoninic acid protein assay (Pierce Chemical Co., USA). Thirty micrograms of total cellular protein was separated by 12% SDS-polyacrylamide gel electrophoresis and then transferred to nitrocellulose membranes. Blots were probed with an antibody specific for the following proteins: *β*-actin (1 : 5000 dilution; Assay Designs, USA); phospho-ERK (p-ERK) and total ERK (1 : 1000 dilution; Cell Signaling Technology, USA); phospho-Akt (p-Akt) and total Akt (1 : 500 dilution; Santa Cruz Biotechnology, USA); HO-1 (1 : 1000 dilution; Cell Signaling Technology, USA); Nrf2 (1 : 500 dilution; Santa Cruz Biotechnology, USA); SMAD (1 : 500 dilution; Santa Cruz Biotechnology, USA); and TGF-*β* (1 : 1000 dilution; Abcam, UK). The binding antibody of each blot was evaluated by enhancing chemiluminescence (Western blot detection kit; Amersham Pharmacia Biotech, USA), which was assessed with horseradish peroxidase-conjugated secondary antibody.

### 2.3. Animal Groups and Treatment Protocol

We carried out this experiment strictly following the recommendations in the Guide for the Care and Use of Laboratory Animals of the National Institutes of Health. The protocol was approved by the Institutional Animal Care and Use Committee in the School of Medicine, The Catholic University of Korea.

Sprague-Dawley (SD) male rats aged 8 weeks were randomly divided into 4 groups (8 rats in each group), which were submitted to (1) sham operation only (normal control), (2) androgen deprived only (Androgen-dep. control), (3) androgen deprived treated by KH-204 200 mg/kg (Androgen-dep. 200), and (4) androgen deprived treated by KH-204 400 mg/kg (Androgen-dep. 400). We administered either distilled water (sham operated) or leuprorelin 0.5 mg/kg subcutaneously once to the backs of androgen-deprived rats. According to previous experimental results, we selected the dosage of leuprorelin [23]. In each group, once-daily oral administration was lasted for 4 weeks (distilled water or KH-204 dissolved in distilled water). After 4 weeks, the animals in all groups were sacrificed under anesthetic and testes, epididymides, and blood samples were obtained.

### 2.4. Measurement of Serum Testosterone Level

Before the rats were sacrificed, venous blood samples were collected from the inferior vena cava and were assayed by an ELISA testosterone detection kit (BioVendor, Czech Republic) to determine the serum testosterone level.

### 2.5. Testicular Histologic Evaluation and Immunohistochemistry

The fixed and embedded testicular tissues were stained with haematoxylin-eosin, and these were examined under a light microscope. Ten representative sites were selected randomly in seminiferous tubules, and spermatogenic cell density was measured as previously described [24].

Leydig cells were identified by histochemically staining for Leydig cell-specific marker 3*β*-hydroxysteroid dehydrogenase (3*β*-HSD) [25]. On each occasion, tissue sections in each experimental group were immunostained and the intensity of the immunostaining was scored using a simplified scale ranging from negative (−) through weakly positive (+) to intensely positive (+++), as previously described [26].

### 2.6. Measurement of Oxidative Stress

Oxidative stress was assessed by measuring the 8-hydroxy-2-deoxyguanosine (8-OHdG) content and superoxide dismutase (SOD) activity quantitatively. Total DNA was extracted from the testis using the DNeasy Blood & Tissue kit (Qiagen, Valencia, CA, USA). The level of 8-OHdG was measured with a DNA oxidation kit (Highly Sensitive 8-OHdG Check ELISA; Japan Institute for the Control of Aging, Fukuroi, Japan). After the final color was developed with the addition of 3,3′,5,5′-tetramethylbenzidine, absorbance was measured at 450 nm. Tissue sample concentration was measured from a standard curve and corrected for DNA concentration. SOD activity (CuZnSOD and Mn SOD) in tissues was determined using an SOD Assay Kit-WST (Dojindo), and the decrease in the rate of the superoxide-mediated reduction of nitroblue tetrazolium was monitored at 450 nm using a spectrophotometer.

### 2.7. Assessment of Apoptosis

Testicular tissue sections were washed out with PBS after blocking with 0.1% Triton X-100 last 5 min. Terminal deoxyribonucleotidyl transferase-mediated dUTP-digoxigenin nick-end labeling (TUNEL, ApopTag In Situ Apoptosis Detection Kits; Millipore, MA, USA) detection solution was dropped on each section and then incubated at 37°C in the dark which lasted for an hour. Nuclear staining with DAPI was performed last for 5 min after being washed out with PBS, and the sections were fixed with 50% glycerol after being washed out with PBS. For the control sections, the TUNEL solution was replaced with PBS. The sections were observed under a fluorescence microscope.

### 2.8. Western Blot Testing *In Vivo*

We gathered testicular tissue proteins of each group, by placing crushed testicular tissues in an ice-cold lysis buffer consisting of 0.1% sodium dodecyl sulfate in phosphate-buffered saline, followed by brief sonication. Protein concentration was identified by a bicinchoninic acid protein assay (Pierce Chemical Co., USA). Thirty micrograms of total testicular tissue protein was separated by 12% SDS-polyacrylamide gel electrophoresis and then transferred to nitrocellulose membranes. Blots were probed with an antibody specific for the following proteins: *β*-actin (1 : 5000 dilution; Assay Designs, USA); HO-1 (1 : 1000 dilution; Cell Signaling Technology, USA); Nrf2 (1 : 500 dilution; Santa Cruz Biotechnology, USA); SMAD (1 : 500 dilution; Santa Cruz Biotechnology, USA); and TGF-*β* (1 : 1000 dilution; Abcam, UK). The binding antibody of each blot was evaluated by enhancing chemiluminescence (Western blot detection kit; Amersham Pharmacia Biotech, USA), which was assessed with horseradish peroxidase-conjugated secondary antibody.

### 2.9. Statistical Analysis

Statistical analyses were carried out using SPSS 16.0 (SPSS Inc., Chicago, USA). The data was expressed as mean ± standard deviation. Statistical significance was analyzed by the ANOVA test, with group comparisons made by Scheffe's test. *p* < 0.05 was considered significant.

## 3. Results

### 3.1. KH-204 Protected TM3 Leydig Cells against Oxidative Stress via Decreased Expression of TGF-*β*1/SMAD and Increased Nrf2/HO-1 Expression

**Figure 1:**
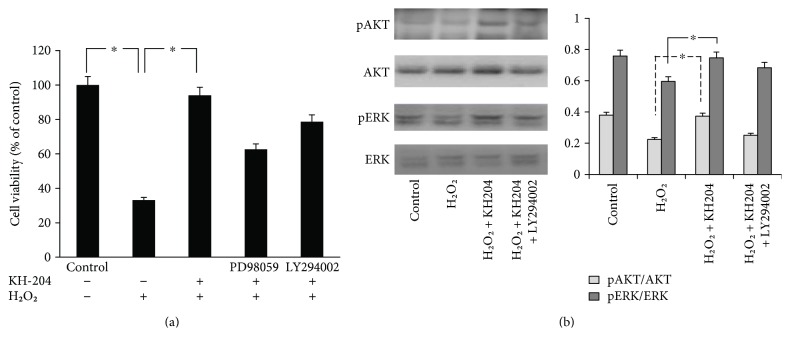
(a) Protective effect of KH-204 against oxidative stress. (b) Enhanced activation of ERK and Akt by KH-204 after 24 h of treatment. The difference was statistically significant (^∗^*p* < 0.05). And the experiments were repeated for three times.

As shown in Figure 1(a), cell viability significantly decreased under the H_2_O_2_ incubation, compared to the cells in none H_2_O_2_ condition (*p* < 0.05). Cell viability was raised to 94% by the addition of KH-204 but was slightly decreased by pretreatment with the ERK inhibitor PD98059 or the Akt inhibitor LY294002.

**Figure 2:**
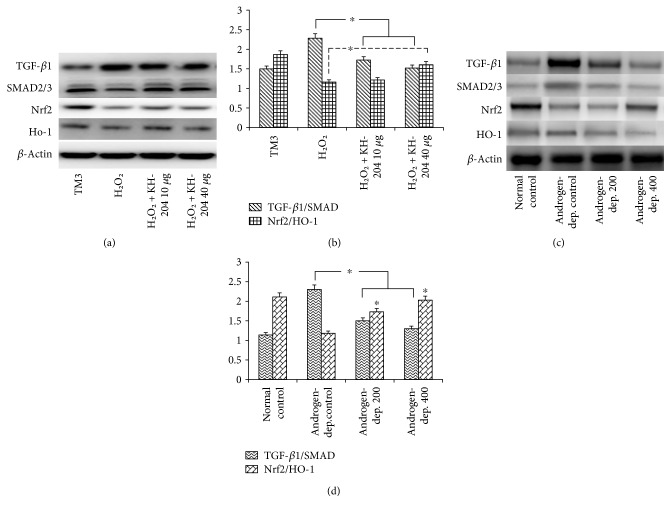
Comparison of the expression levels in vitro and in vivo. (a) is TGF-*β*1/SMAD and Nrf2/HO-1 in vitro, (b) is densitometric analysis relative to *β*-actin in vitro, (c) is TGF-*β*1/SMAD and Nrf2/HO-1 in vivo, and (d) is densitometric analysis relative to in vivo. The difference was statistically significant (^∗^*p* < 0.05). And the experiments were repeated for three times.

Western blot analysis was used to assess whether KH-204 could keep TM3 cells from H_2_O_2_-induced damage. The phosphorylation levels of ERK and Akt were significantly upgraded by KH-204 treatment (*p* < 0.05, Figure 1(b)). However, treatment with the Akt inhibitor LY294002 decreased the phosphorylation levels by inhibiting PI3K in the treatment group. These results showed that KH-204 could effectively protect against H_2_O_2_-induced damage via ERK and Akt activation. The quantitative result showed that expression of TGF-*β*1/SMAD was significantly increased and Nrf2/HO-1 was meaningfully decreased after H_2_O_2_ injury (*p* < 0.05, Figure 2(b)). And this result illuminated that the effect of recovery was according to the dose of KH-204, which means the result is better in the H_2_O_2_ + KH-204 40 *μ*g group than in the H_2_O_2_ + KH-204 10 *μ*g group. In the animal experiment, the Western blot results (Figures 2(c) and 2(d)) showed that expression of TGF-*β*1/SMAD was significantly increased and Nrf2/HO-1 was meaningfully decreased after treatment by leuprorelin (*p* < 0.05). However, with treatment of KH-204, the results of protein expression significantly changed, which turned to normality (*p* < 0.05, Figures 2(c) and 2(d)).

### 3.2. KH-204 Preserved Testicular Function and Serum Testosterone Levels in an *In Vivo* Model

There were no significant differences in body weight among the test groups. In Table 1, the mean weights of the testis of each group and the epididymis are listed. There was a sensible difference in the testicular and the epididymal weights between the normal control group and the androgen-deprived control group after 4 weeks (*p* < 0.05). However, treatment by KH-204 caused obvious increases in testicular and epididymal weights in the androgen-deprived 400 mg/kg group when compared with those in the androgen-deprived control group (*p* < 0.05).

**Figure 3:**
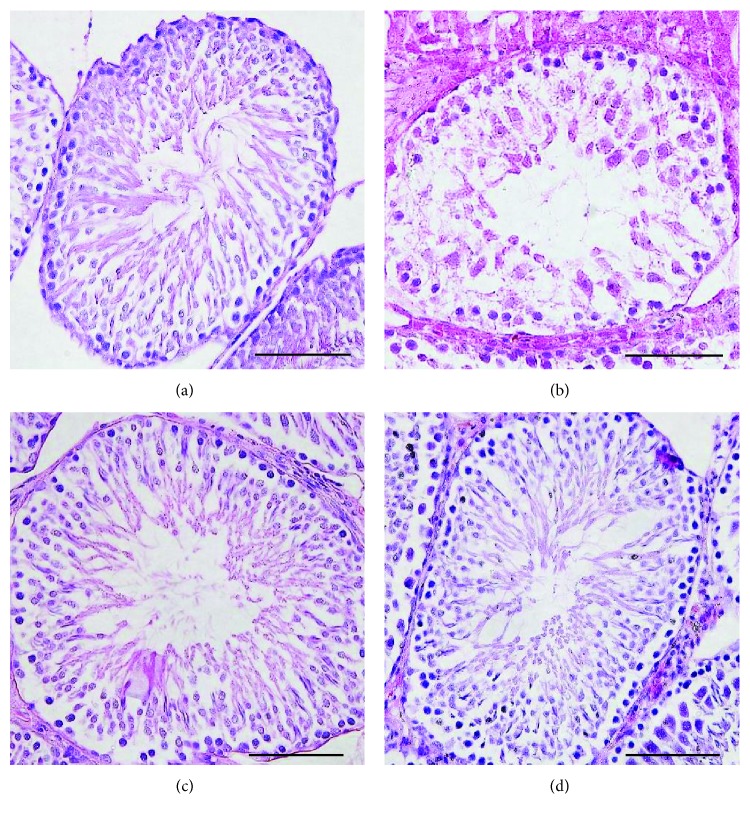
Histopathological findings of the testicular tissues (haematoxylin and eosin stain) in (a) the normal control group (*n* = 8, normal conrtol), (b) the androgen-deprived control group (*n* = 8, Androgen-dep. control), (c) the androgen-deprived 200 mg/kg group (*n* = 8, Androgen-dep. 200), and (d) the androgen-deprived 400 mg/kg group (*n* = 8, Androgen-dep. 400). Compared with the normal control group, a narrow germinal cell layer is observed in the androgen-deprived control group. Scale bars shown in each figure represent 100 *μ*m. And the experiments were repeated for three times.

The testicular tissues of all groups showed normal structure with mature seminiferous tubules and complete spermatogenic series. However, the spermatogenic cell densities of the testes in the androgen-deprived control group were slightly reduced in comparison with those in the other 3 groups (Figure 3). The tissue was degenerated, and incomplete spermatogenic series were shown in some seminiferous tubules. There were almost normal mature active seminiferous tubules with complete spermatogenic series in the treatment group. The mean thickness of the germinal cell layer and the mean diameter of the seminiferous tubules in the androgen-deprived 400 mg/kg group were significantly increased in comparison with those in the androgen-deprived control group (Table 1).

The serum testosterone levels in the androgen-deprived control group were obviously decreased in comparison with those in the normal control group (*p* < 0.05, Table 1). These values were dose dependently increased after KH-204 treatment, and those from the androgen-deprived 400 mg/kg group were significantly increased compared with those from androgen-deprived control group (*p* < 0.05).

### 3.3. Immunohistochemistry

**Figure 4:**
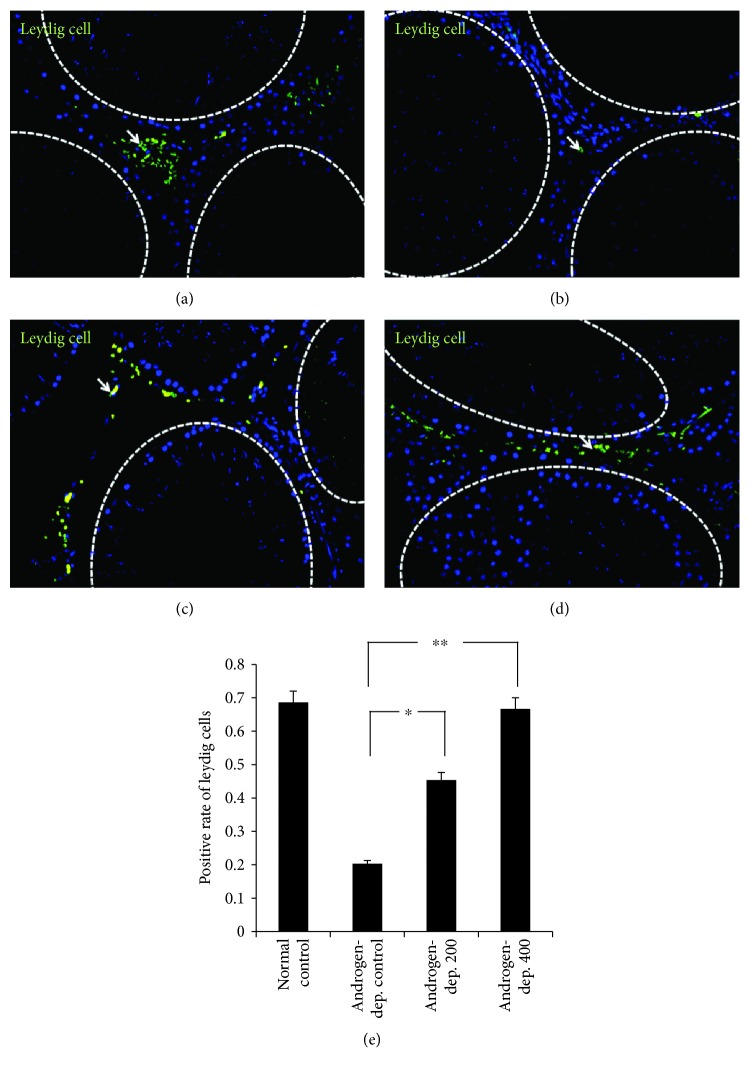
Immunoexpression of 3*β*-HSD (arrow) in Leydig cells of rat testis after treatment. ×400. (a) The normal control group (*n* = 8, normal control); (b) the androgen-deprived control group (*n* = 8, Androgen-dep. control); (c) the androgen-deprived 200 mg/kg group (*n* = 8, Androgen-dep. 200); (d) the androgen-deprived 400 mg/kg group (*n* = 8, Androgen-dep. 400); and (e) positive rate of 3*β*-HSD-immunoreactive cells. The difference was statistically significant (^∗^*p* < 0.05 and ^∗∗^*p* < 0.01). And the experiments were repeated for three times.

Cells positive for 3*β*-HSD activity are shown in Figures 4(a), 4(b), 4(c), and 4(d). They can be seen in the testicular interstitium, and the intensity was significantly decreased after androgen deprivation. However, the number of 3*β*-HSD-immunoreactive cells was relatively increased after KH-204 treatment (*p* < 0.05, Figure 4(e)). Moreover, the intensity in the androgen-deprived 400 mg/kg group was comparable to that in the normal control group.

### 3.4. KH-204 Decreased Oxidative Stress and Apoptosis

**Figure 5:**
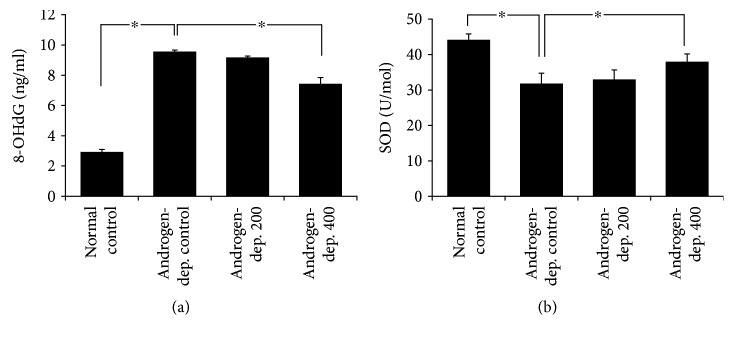
Comparison of the expression levels of 8-OHdG (a) and SOD (b). The difference was statistically significant (^∗^*p* < 0.05) (*n* = 8, in each group). And the experiments were repeated for three times.

**Figure 6:**
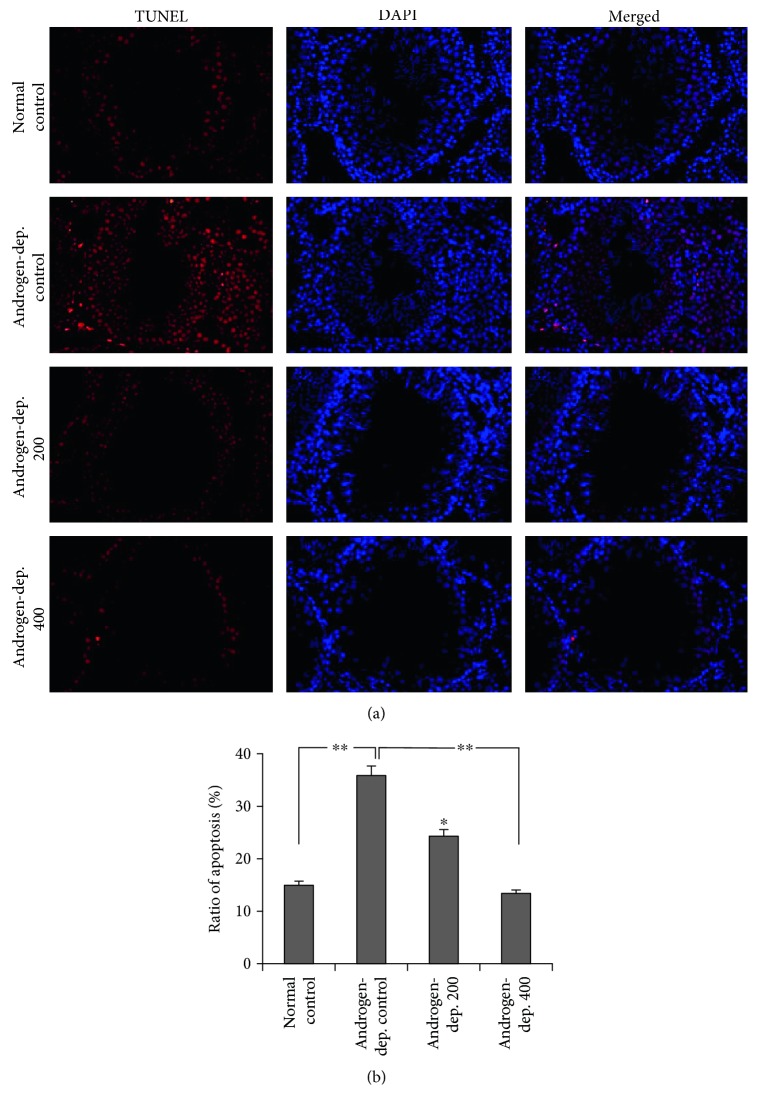
Effect of KH-204 on testicular tissue apoptosis by TUNEL assay. The difference was statistically significant (^∗^*p* < 0.05 and ^∗∗^*p* < 0.01) (*n* = 8, in each group). And the experiments were repeated for three times.

The mean expression of 8-OHdG and SOD is shown in Figure 5. After treatment by KH-204, a dose-dependent decrease in oxidative stress was found. Oxidative stress significantly increased in the androgen-deprived control group in comparison with that in the normal control group but obviously reduced in the androgen-deprived 400 mg/kg group after treatment (*p* < 0.05). The apoptotic cells in the testis were observed as being dark red in color in the TUNEL assay (Figure 6). The increased apoptotic cells in the androgen-deprived control group were significantly reduced in the two treatment groups receiving a dose of KH-204 (*p* < 0.05).

## 4. Discussion

The present study showed that KH-204 increased the viability of TM3 cells in oxidative-stressed conditions. LOH that bothers many middle-aged males is mainly caused by serum levels of testosterone that decline with age. In the aging male, serum testosterone continues to decrease because of reduced function of Leydig cells [27]. Although the age-related mechanism which could induce decreased function of Leydig cells remains unclear, our data suggest that there is a possibility to change the redox balance of the Leydig cells [28]. In this study, we showed that KH-204 increased the viability of TM3 cells under oxidative-stressed conditions in vitro and in vivo, which means KH-204 can protect the Leydig cells against damage from oxidative stress response. What is more, we detected the testicular function, serum testosterone levels, oxidative stress, and apoptosis in androgen-deprived rats, and these results illuminated that KH-204 could improve the survival of Leydig cells under androgen-deprived conditions in vivo.

Some researchers studied the shifts in Nrf2/electrophile response element activity in older organisms. Suh et al. [29] showed that protein expression of Nrf2 was reduced obviously, combined with a decreased GCL expression. The similar results were also observed in another study, which nuclear Nrf2 was reduced in the aorta of elder groups, along with reduced glutamate cysteine ligase, NADPH:quinone oxidoreductase-1, and HO-1 levels [30]. Although there is no report on the shifts in the expression levels of HO-1 with older testis, we found that the levels of Nrf2/HO-1 were obviously reduced after oxidative stress and ameliorated with the treatment of KH-204 in TM3 Leydig cells.

To examine if KH-204 can increase androgen synthesis in the androgen-deprived rat, we analyzed influences of KH-204 on the contents of serum testosterone and 3*β*-HSD activities. The present data show that the testosterone concentration and the number of 3*β*-HSD-immunoreactive cells were significantly increased after treatment with KH-204, suggesting a protective effect for KH-204. These findings also support the possibility that an increase in serum testosterone might be induced by the protective actions of KH-204 on Leydig cells which could express testosterone in the testis. KH-204 simultaneously increased spermatogenesis and the germinal cell layer thickness. The active sperm production was influenced by the contents of serum testosterone which could upgrade the efficiency of differentiation and active maturation in germ cell [31]. Therefore, consistent with results from previous studies, we suggest that the improved activity of Leydig cells along with the increased serum testosterone levels by KH-204 might improve spermatogenesis.

Aging is along with reproductive dysfunction where there are significant declines in steroidogenesis, spermatogenesis, and sexual function that might be due to the degradation of testicular function [32, 33]. Although the aging mechanism is confusing, there are commonly held beliefs that two major factors in aging are oxidative stress and apoptosis [34, 35]. Lesniewski et al. [36] found that oxidative stress in cells gradually increased with age in mice. Apoptosis is defined as a kind of biochemical and morphological change in various cellular levels, leading to the removing of unnecessary cells. It is an important physiological process [37]. However, apoptosis also has an unwanted influence on aging as it often eliminates important cells that are related to aging [38]. We observed that levels of 8-OHdG degraded and levels of SOD upgraded and that apoptotic cells were significantly decreased following treatment with KH-204 compared with the androgen-deprived group. KH-204 may alleviate testicular dysfunction in androgen-deprived rats via suppression of oxidative stress and apoptosis.

Recent data suggested that there might be a joint pathway for these different tissue damages, which was known as the upregulation of TGF-*β*1/SMAD signaling pathway [20, 39]. As is known to all, TGF-*β* can intermediate its fibrotic effects by triggering the receptor-associated SMADs [40]. Although there is no report on the effect of TGF-*β*1/SMAD pathway on the testis, many reports investigated that it might play a key role in configurational changes for erection [41, 42]. In our study, the expression of TGF-*β*1/SMAD was higher after H_2_O_2_ injury, and these proteins' contents were obviously reduced in the KH-204 treatment groups in comparison with the group without KH-204. It is likely that KH-204 is at least in part attributable to the antifibrotic effect in the testis.

Herbs have been used widely in various urologic diseases, and many researches have been carried out to prove their safety and efficacy [43]. In particular, herbal medicines such as KH-204 are more essential for the treatment of LOH due to the adverse effects of ART. Discovery of natural products or herbs that can protect Leydig cells and restore the production of serum testosterone is very important to improve LOH. Previous studies demonstrated that KH-204 ameliorated ED in aged and diabetic rats [10, 11]. We also found that KH-204 protected against oxidative stress in the cryptorchid testis [12]. However, few studies on the detailed mechanisms against LOH have been reported. The major ingredients in KH-204 were reported to have antioxidant effects in various diseases. Furthermore, it was shown in a previous study that *Cuscuta chinensis* Lam. might improve kidney yang deficiency symptoms by recovering decreased serum testosterone [44]. In this study, we identified that KH-204 treatment improved the viability in oxidative stressed TM3 cells and that the activation of the ERK/Akt-dependent signaling pathways is the main mechanism. Thereafter, we illuminated that treatment with KH-204 can reduce oxidative stress or apoptosis in the androgen-deprived rat model.

Our study does have some limitations. First, the model used is far from a definite model of LOH. Aged or castrated animals have commonly been used as LOH animal models in many studies for oral supplementation [45, 46]. However, we wanted to reproduce a partial androgen deficiency state rather than an entire castration, and low-dose leuprorelin injection was useful for creating this situation. Secondly, while we clarified antioxidant and antifibrotic functions for KH-204, we did not identify the assumed mechanism, through the animal model. In the next work, we should try our best to investigate the accurate mechanism of KH-204 in the LOH animal model to plan for a clinical study.

We studied the efficacy of KH-204 to improve testicular dysfunction in LOH. The efficacies of KH-204 are likely, at least in part, to degrade oxidative stress through the Nrf2/HO-1 pathway. These findings may offer credible evidences for the use of new alternative therapies to treat LOH.

## Figures and Tables

**Table 1:** Comparisons of parameters of the testicular health.

	Testicular weight (g)	Epididymal weight (g)	Germinal cell layer thickness (*μ*m)	Diameter of seminiferous tubules (*μ*m)	Serum testosterone (ng/ml)
Normal control	1.62 ± 0.19	0.68 ± 0.03	73.22 ± 6.34	297.34 ± 4.33	2.48 ± 0.41
Androgen-dep. control	1.09 ± 0.06^∗^	0.40 ± 0.08^∗^	42.19 ± 2.69^∗^	251.16 ± 3.62^∗^	1.28 ± 0.34^∗^
Androgen-dep. 200	1.15 ± 0.17	0.45 ± 0.06	52.46 ± 3.92	271.31 ± 1.36	1.32 ± 0.52
Androgen-dep. 400	1.30 ± 0.05^∗∗^	0.59 ± 0.01^∗∗^	69.86 ± 7.10^∗∗^	280.14 ± 8.32^∗∗^	1.72 ± 0.13^∗∗^

Data show the mean ± s.d. analysis of variance test. ^∗^Significant statistical difference (*p* < 0.05) compared with the normal control group. ^∗∗^Significant statistical difference (*p* < 0.05) compared with the androgen-deprived control group.
